# Deleterious Mutations in DNA Repair Gene *FANCC* Exist in *BRCA1/2*-Negative Chinese Familial Breast and/or Ovarian Cancer Patients

**DOI:** 10.3389/fonc.2019.00169

**Published:** 2019-03-22

**Authors:** Zhi-Wen Pan, Xiao-Jia Wang, Tianhui Chen, Xiao-Wen Ding, Xiyi Jiang, Yun Gao, Wen-Ju Mo, Yuan Huang, Cai-Jin Lou, Wen-Ming Cao

**Affiliations:** ^1^Department of Clinical Laboratory, Zhejiang Cancer Hospital, Hangzhou, China; ^2^Department of Breast Medical Oncology, Zhejiang Cancer Hospital, Hangzhou, China; ^3^Group of Molecular Epidemiology & Cancer Precision Prevention (GMECPP), Zhejiang Academy of Medical Sciences (ZJAMS), Hangzhou, China; ^4^Department of Breast Cancer Surgery, Zhejiang Cancer Hospital, Hangzhou, China; ^5^Institute of Cancer Research, Zhejiang Cancer Hospital, Hangzhou, China

**Keywords:** Chinese, familial breast cancer, familial ovarian cancer, *FANCC*, deleterious mutation, susceptibility

## Abstract

**Introduction:** *FANCC* is reported as a novel susceptibility gene for breast cancer, however, its mutation remains unclear in Chinese population. We aimed to identify the germline mutations of *FANCC* in high-risk breast cancer patients in China.

**Methods:** 255 *BRCA1/2*-negative Chinese familial breast and/or ovarian cancer (FBOC) patients were recruited for *FANCC* germline mutations screen. For whom 90 patients were detected by PCR-sequencing assay, and another 165 patients were detected by a 98-gene panel sequencing assay. The 98-gene panel sequencing assay was also used to screen other possible gene mutations for the patients with *FANCC* mutations detected by PCR-sequencing assay. Two hundred and fifty sporadic breast cancer (SBC) patients and 248 female non-cancer controls (FNCCs) were recruited for the genotyping analysis. Immunohistochemistry (IHC) analysis was used to evaluate the FANCC expression in patients with *FANCC* mutation.

**Results:** We found one rare *FANCC* deleterious mutation (c.339G>A, p.W113X, 0.4%) and two novel non-synonymous variants (c.51G>C, p.Q17H, 0.4% and c.758C>A, p.A253E, 0.4%) in FBOC patients, whereas none of above mutations was identified in SBC patients or FNCCs. We also found that one novel synonymous variant (c.903A>G, p.A301A) existed in one FBOC patient. Additionally, two non-synonymous SNPs rs201407189 (c.973G>A, p.A325T) and rs1800367 (c.1345G>A, p.V449M), and two synonymous SNPs rs55719336 (c.816C>T, p.I272I) and rs79722116 (c.1407G>A, p.T469T) were identified in FBOC patients.

**Conclusion:** *FANCC* deleterious mutations exist in Chinese FBOC patients and investigations on the penetrance and spectrum of *FANCC* mutations need to be further conducted.

## Introduction

Breast cancer is the most common malignancy in Chinese women, which accounts for 11.2% newly diagnosed cases and 9.2% deaths from breast cancer worldwide (1). The onset age is ~ 10 years younger in Chinese women compared to women in western countries (2). The study from our group (3), summarizing the characteristics of germline mutations in breast cancer susceptibility genes in Chinese women with high-risk breast cancer, found that *BRCA1/2* mutations accounted for the majority of hereditary breast cancer, while other genes such as *TP53, BRIP1, PALB2, CHEK2, RAD50, NBS1*, and *RAD51C* were only responsible for a smaller fraction (3). However, the genetic etiology for more than 80% Chinese women with high-risk breast cancer still remains unknown.

Increasing efforts have been invested to identify novel susceptibility genes that predispose individuals to breast cancer. Several rare moderate-penetrance susceptibility genes including *XRCC2*, (4) *BLM*, (5) *FANCC*, (5) *RECQL*, (6) *MCPH1*, (7), and (8) are identified by the next-generation sequencing assay. Thompson et al. (5) found four found four deleterious mutations in DNA repair gene *FANCC* in 1,410 breast cancer families. Among these mutations, two truncating mutations were found in 15 *BRCA1/2*-negative high-risk breast cancer families by whole-exome sequencing, and another two were found in 438 validating *BRCA1/2*-negative breast cancer families screened over the entire coding region by Sanger sequencing. In addition, one mutation in additional 957 *BRCA1/2* uninformative breast cancer families was found through the mutation hotspot screening. However, none of these mutations were identified either in the healthy controls or in the 1,000 Genomes Project database. These results suggested that deleterious mutations in *FANCC* gene play a role in breast cancer predisposition.

Investigations on the germline mutation of *FANCC* in high-risk breast cancer patients are sparse. Therefore, we aimed at, for the first time in mainland China, assessing the germline mutation of *FANCC* in high-risk breast cancer patients in women from eastern China, by screening the complete coding regions and exon-intron boundaries of *FANCC*.

## Materials and Methods

### Patient/Sample Ascertainment

The eligibility criteria for this study were breast cancer patients who had at least one first- or second-degree relative affected with breast cancer and/or ovarian cancer, regardless of the diagnosis age. A total of 335 unrelated breast cancer patients fulfilled this criterion. All of the participants were ascertained between the years 2008 and 2018 in the Zhejiang Cancer Hospital in Zhejiang, an eastern province of China. Small mutations in *BRCA1/2* were analyzed in 133 patients using polymerase chain reaction (PCR)-sequencing assay and also in another 202 patients using 98-gene panel sequencing assay. Large genomic rearrangements (LGRs) in *BRCA1/2* were analyzed in all 335 patients using a multiplex ligation-dependent probe amplification (MLPA) assay. Among them, we found 73 breast cancer patients carrying *BRCA1*/*2* small mutations and seven patients carrying *BRCA1* LGRs (9, 10). Finally, blood samples from 255 unrelated *BRCA1/2*-negative breast cancer patients were enrolled for DNA sequencing in this study. Additionally, 250 sporadic breast cancer (SBC) patients and 248 female non-cancer controls (FNCCs) were selected for genotyping analyses in the Zhejiang Cancer Hospital. The use of tissue samples in this study was approved by the Research and Ethical Committee of the Zhejiang Cancer Hospital. All experiments were performed in accordance with the approved guidelines. Written informed consent was obtained from all participating patients prior to clinical data and peripheral blood collection. Peripheral blood samples were collected from each proband and from as many affected relatives as possible. All of the blood samples were collected in EDTA tubes and stored at −80°C.

### *FANCC* Mutation Analysis

Genomic DNA was prepared from peripheral blood leukocytes from the proband of each family using the QIAamp DNA Blood Mini kit (Qiagen, Hilden, Germany). The complete coding regions and exon-intron boundaries of *FANCC* [NM_000136.2] were screened in the first phase for 90 patients using a PCR-sequencing assay, and another 165 patients were also screened for mutations of *FANCC* using a 98-gene panel sequencing assay. Furthermore, the patients found with *FANCC* deleterious mutations in the first phase were further screened for mutations in other 97 genes by the 98-gene panel sequencing assay.

The primers for PCR-sequencing assay were designed using Primer Premier 5.0 (Premier, CA). Overall 14 pairs of primers for amplifying the whole coding sequences and their flanking sequences in introns were synthesized by Invitrogen (Invitrogen, Carlsbad, CA, USA). Information on the primers was listed in Table 1. The reaction conditions were as follows: an initial denaturation at 94°C for 5 min, followed by 30 cycles of denaturing at 94°C for 30 s, annealing at 60°C for 30 s, and the extension at 72°C for 60 s; finally, the reaction was elongated at 72°C for 5 min. All fragments were sequenced using a BigDye Mix and an ABI 3730xl Genetic Analyzer (Applied Biosystems, Foster City, CA), and the data were analyzed by Mutation Surveyor (Softgenetics Inc., USA). Each mutation was confirmed by duplicate independent PCR-sequencing assays. All variants were named according to the Human Genome Variation Society (HGVS) sequence systematic nomenclature (http://www.hgvs.org/mutnomen/). Mutalyzer Name Checker (http://mutalyzer.nl) was used to check variant descriptions.

**Table 1:** Primers for entire coding exons and intron-exon boundaries of *FANCC*.

**Exons**	Primer sequence (5′-3′)^*^	**Product size (bp)**
Exon1	F: ATAATTAGCGTGTGCCTGTGGA	722
	R: ACTTGCTTGGTCAGGAAGTGT	
Exon2	F: TGGAGCTGAGTTCGTAACCTCT	972
	R: CAAAGTCACGGCAGGATTCAC	
Exon3	F: AGTGATCCCAAGGCCACAAG	742
	R: AGGCATGGAAGCATGTGGAA	
Exon4	F: GCCAAGCCTCTTCCCTGATG	995
	R: GCCATAAGTCTGCCCAAGGT	
Exon5	F: GTTGGGGGAATCTCAACGGA	561
	R: CAGAAGAAGGCAGAGCCAGG	
Exon6	F: TTGGGCCTGAGCAAACAAGA	564
	R: TTTCCAACACACCACAGCCT	
Exon7	F: TTAGGGACTGGGCATCACGA	973
	R: AGGTGGCCTCACACCAAAAG	
Exon8	F: GCCAGTTTTCTGGACATCAGC	992
	R: ACCCCCAACACTGTTCTGAC	
Exon9	F: TCTAGCCCCTCCCACCTAAC	880
	R: AACCTTTGTTGGGGCACTCA	
Exon10	F: GGGCAGAGGACTCAGAGTTTTG	860
	R: CCCATGTCAGGACTGCCTTC	
Exon11	F: GAAACCTAGGGCATCCGCAA	821
	R: GGTCCCAGACCAGTAATGCC	
Exon12	F: CCACATCCTGCCACATTCCT	683
	R: GAGAACGCCTCTGACCACAA	
Exon13	F: AGTGCTTACCCGTTTCTGGG	674
	R: CCCATTCTCATCGTGGCCTT	
Exon14	F: GTGGTTATGGTCCGTCCCTG	829
	R: AGAGTGGAAAGAGTGTGCCG	

* *F, forward; R, reverse*.

A 98-gene panel was designed using the NEBNext direct sequencing technology conducted by New England Biolabs (Ipswich, MA). The panel contains 98 genes including 24 known and candidate breast cancer susceptibility genes (*ATM, BARD1, BLM, BRCA1, BRCA2, BRIP1, CDH1, CHEK2, FANCC, FANCM, FANCR/RAD51, FANCU/XRCC2, MCPH1, MRE11A, NBN, NF1, PLAB2, PETN, RAD50, RAD51C, RAD51D, RECQL, STK11*, and *TP53*), 48 other cancer susceptibility genes (*ALK, APC, AXIN2, BAP1, BMPR1A, CASR, CDC73, CDK4, CDKN2A, CEBPA, DICER1, EPCAM, FLCN, GATA2, GPC3, HRAS, KIT, MEN1, MLH1, MSH2, MSH6, MUTYH, MET, NF2, PAX5, PGDFRA, PHOX2B, PMS1, PMS2, POLD1, POLE, PRF1, PRKAR1A, PTCH1, RB1, RET, RUNX1, SDHA, SDHB, SDHC, SDHD, SMAD4, SMARCE1, SUFU, TERT, VHL, WRN, WT1*) and other 26 genes (*ABRAXAS1, AIP, CDKN1B, CDKN1C, DIS3L2, FANCA, FANCB, FANCD2, FANCE, FANCF, FANCG/XRCC9, FANCI, FANCL, FANCP/SLX4, FANCQ/ERCC4, FANCT/UBE2T, FH, MAX, RECQL4, SDHAF2, SMARCA4, SMARCB1, TERC, TMEM127, TSC1* and *TSC2*). The 98-gene panel sequencing assay was performed on 165 *BRCA1/2*-negative unrelated patients and on the patients with *FANCC* deleterious mutations by Hiseq X sequencing (Illumina, CA, USA). A minimum 500x mean coverage was achieved for the aforementioned genes. Variants were called from raw FASTQ data by running through the in-house bioinformatics pipeline. Variants were strictly interpreted according to the Standards and Guideline from American College of Medical Genetics (ACMG), Genomics, and the Association for Molecular Pathology (11).

Whether the sequence variants were previously reported was checked in public databases, including the 1,000 Genomes Browser (http://browser.1000genomes.org/), the Genome Aggregation database (gnomAD, http://gnomad.broadinstitute.org/), the NCBI SNP database (http://www.ncbi.nlm.nih.gov/projects/SNP/), the Leiden Open Variation Database 3.0 (LOVD 3.0, http://databases.lovd.nl/shared/genes/FANCC), the Exome Variant Server (http://evs.gs.washington.edu/EVS/), Online Mendelian Inheritance in Man (OMIM) (http://www.omim.org/), and ClinVar database (https://www.ncbi.nlm.nih.gov/clinvar/).

### *In silico* Prediction

To identify the non-synonymous variants (regardless of whether or not *FANCC* function was disrupted), we conducted *in silico* prediction using four comparative evolutionary bioinformatics programs: SIFT (http://http://sift.jcvi.org/), PolyPhen-2 (http://genetics.bwh.harvard.edu/pph/), PROVEAN (http://provean.jcvi.org/index.php), and PANTHER (http://www.pantherdb.org/tools/csnpScoreForm.jsp). MutationTaster (http://www.mutationtaster.org/) analyses were also conducted for functional prediction.

### Genotyping in Breast Cancer Cases and Non-cancer Controls

Associations between the selected variants and breast cancer were evaluated in further studies. Three variants were genotyped in a set of 250 SBC patients and 248FNCCs. Genotyping of the variants c.339G>A and c.973G>A was performed with the MassARRAY platform (Sequenom, San Diego, CA, USA) using the iPLEX Gold Assay. The amplification and its extended primers were designed by MassARRAY Designer of Sequenom. The information on the primers was listed in Table 2. The amplification reaction conditions were as follows: an initial denaturation at 94°C for 15 min, followed by 45 cycles of denaturing at 94°C for 20 s, annealing at 56°C for 30 s, and the extension at 72°C for 60 s; finally, the reaction was elongated at 72°C for 3 min. Reaction parameters of single-base extension were an initial incubation at 94°C for 30 s, followed by 40 cycles at 94°C for 5 s with 5 nested cycles of 52°C for 5 s and 80°C for 5 s, respectively. Finally, singe-base extension was completed at 72°C for 3 min. Experimental data were analyzed by Typer software version 4.0 (Sequenom, San Diego, CA, USA). Genotyping of the variants c.51G>C and c.758C>A was done by PCR-sequencing assay. The primers and reaction conditions were the same as those used in the mutation screening for *FANCC* gene exon1 and exon7.

**Table 2:** Primers for genotyping analysis by MassARRAY platform.

**Position**	Primers for amplification (5′-3′)^*^	**Primers for single-base extension (5′-3′)**	**Extension direction**
c.339G>A	F: ACGTTGGATGGCAGAGCAAGATTTACTCTC	AGATTTACTCTCTTACCTGTAT	Reverse
	R: ACGTTGGATGAGAACCACAGAATTCTGGAC		
c.973G>A	F: ACGTTGGATGCTATTCAGGTGTTTACGCAG	CGCAGTGCTTTGTAGAA	Reverse
	R: ACGTTGGATGACAGCGTCTTATTCTCTGGG		

* *F, forward; R, reverse*.

### Immunohistochemistry Analysis

The expression of estrogen receptor (ER), progesterone receptor (PR), human epidermal growth factor receptor 2 (HER2), and FANCC was analyzed by immunohistochemistry (IHC) assay in breast cancer tissues. A panel of antibodies was used in this study: anti-ER (clone: SP1 from Roche, #05278406001), anti-PR (clone: 1E2 from Roche, #05277990001), anti-HER-2 (clone: 4B5 from Roche, #05999570001), and anti-FANCC (GeneTex #GTX100400, dilution 1:400). ER and PR positive were defined as ≥1% of tumor cells showing positive nuclear staining. HER2 testing was performed according to the guideline of the American Society of Clinical Oncology/College of American Pathologists for HER2 testing in breast cancer (12). HER2 positive was defined as membrane staining with a score of 3+; when the score ranged 2–3, a fluorescence *in situ* hybridization (FISH) assay was performed to confirm the HER2 status. FANCC displayed cytoplasmic staining. The scores of FANCC expression were classified as below: 0 for no staining; 1 for weak staining; 2 for moderate staining; and 3 for strong staining; staining score ≥1 was considered positive. All immunostains were assessed independently by three pathologists from Zhejiang Cancer Hospital, Hangzhou, China.

### Statistical Analysis

Data were analyzed using the SPSS 17.0 statistical package (SPSS, Chicago, IL, USA). The genotype frequencies of variants in SBC cases and FNCCs were compared using a χ^2^ test. Two-side *P* < 0.05 was considered statistically significant.

## Results

**Figure 1:**
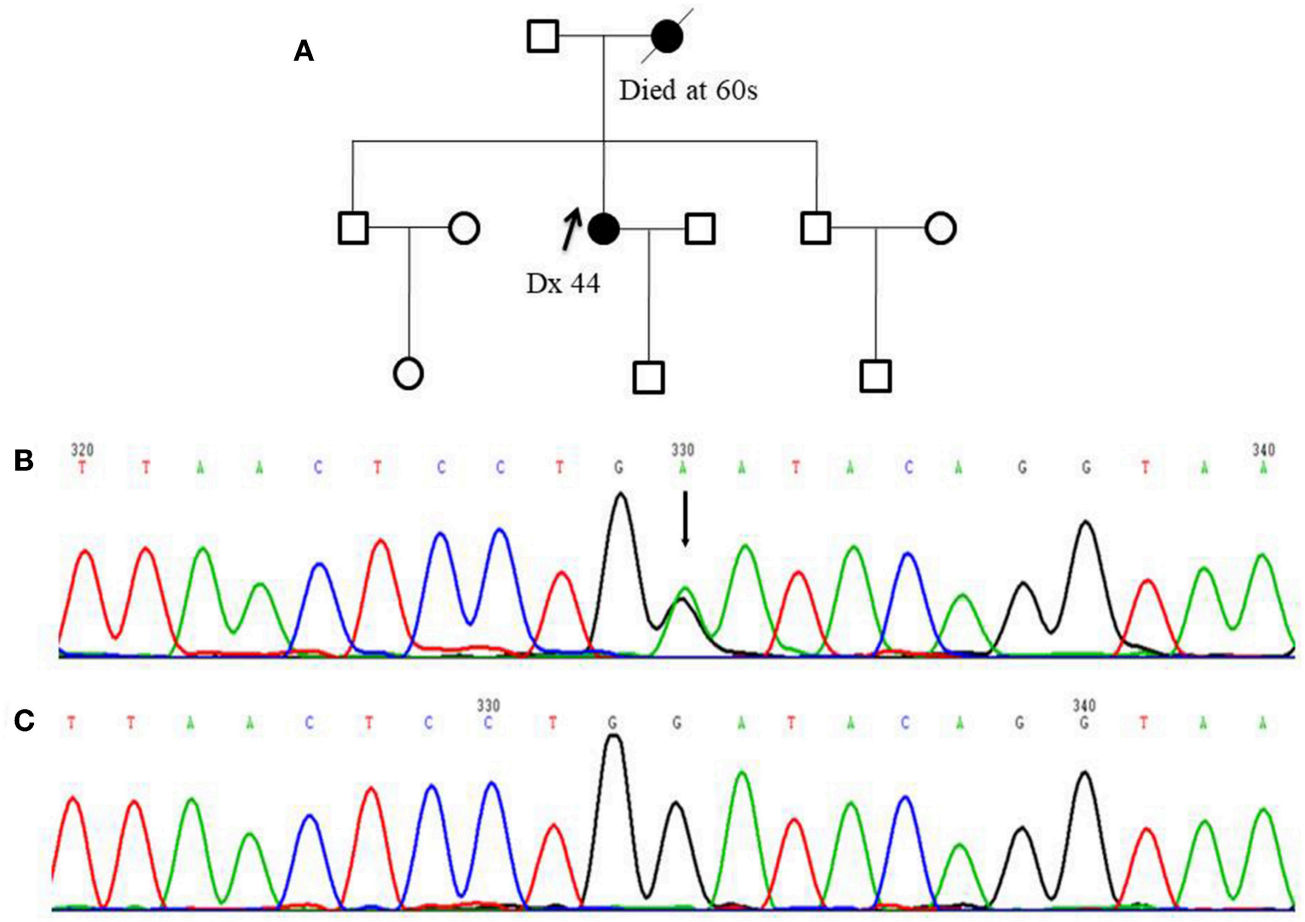
Pedigree of one *FANCC* deleterious mutation case and an electropherogram for the proband with the identified mutation: **(A)** The proband of the family carrying *FANCC* c.339G>A mutation (indicated by black arrow) **(B)** The electropherogram of *FANCC* c.339G>A mutation (affected base is indicated by black arrow) **(C)** Sequence matching wildtype.

**Figure 2:**
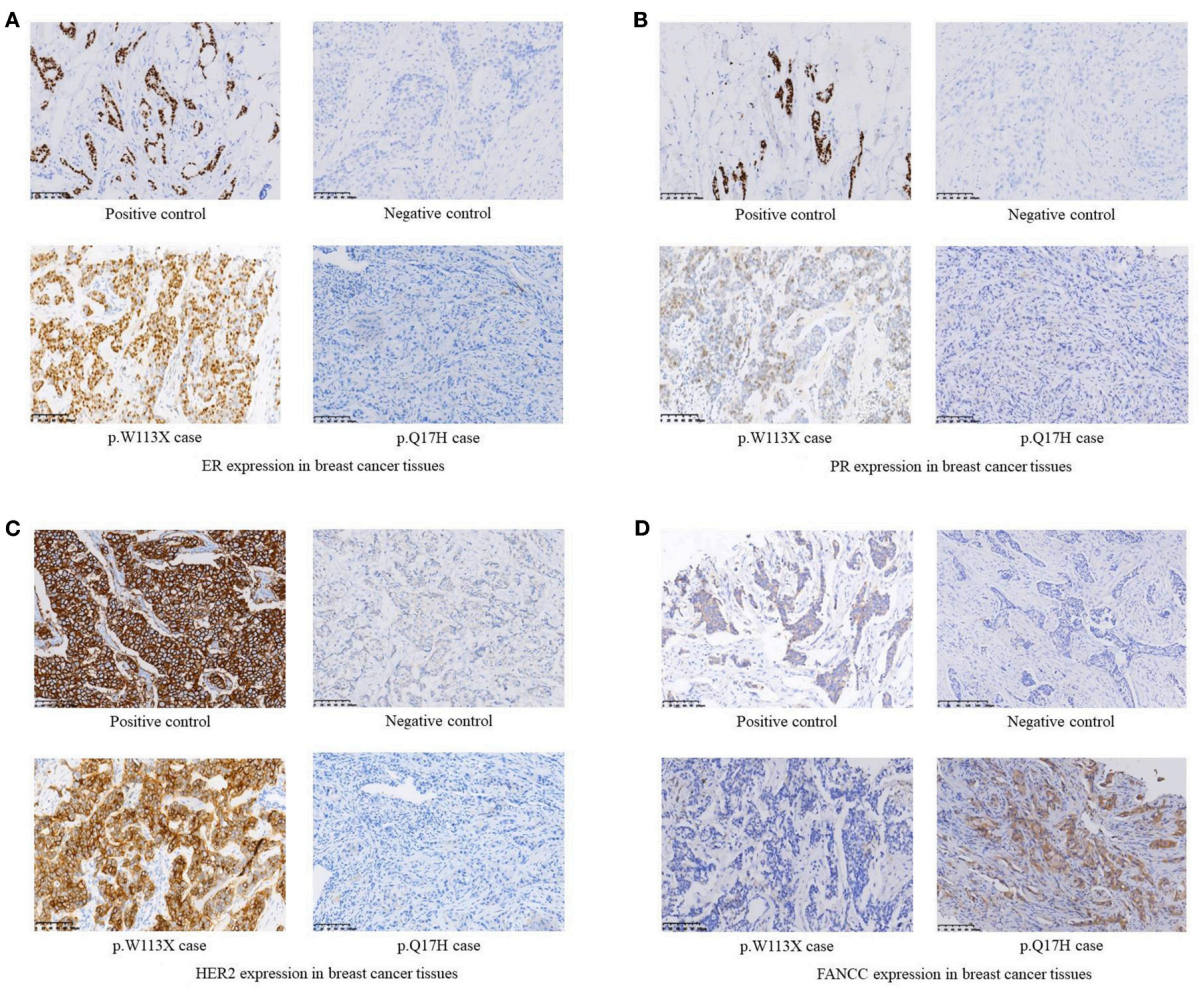
**(A)** ER expression in breast cancer tissues. **(B)** PR expression in breast cancer tissues. **(C)** HER2 expression in breast cancer tissues. **(D)** FANCC expression in breast cancer tissues.

Among 255 familial breast and/or ovarian cancer (FBOC) patients without *BRCA1/2* mutation, we found one (0.4%) patient carrying a non-sense mutation (c.339G>A, W113X), which was also observed in an individual with hereditary cancer-predisposing syndrome, classified as pathogenic/likely pathogenic in ClinVar database (https://www.ncbi.nlm.nih.gov/clinvar/). This mutation was not reported in other public databases, including PubMed (https://www.ncbi.nlm.nih.gov/pubmed/), 1,000 Genomes Browser, NCBI SNP database, LOVD 3.0, and Exome Variant Server. Moreover, this mutation was found in neither 250 SBC cases nor 248 FNCCs in our cohort (Table 3). The pedigree of the mutation carrier and electropherogram for the proband are presented in Figure 1. This patient with *FANCC* c.339G>A mutation was diagnosed with invasive ductal breast cancer at the age of 44 years. IHC analysis showed positive results for the patient's ER, PR, and HER2 while showing a negative result for FANCC (Figure 2). The patient's mother also had breast cancer whereas blood samples from her mother were not available for the mutation analyses. The 98-gene panel sequencing assay identified 195 germline variants including *FANCC* c.339G>A (Supplementary Table 1). None of these variants except *FANCC* c.339G>A could be classified as pathogenic or likely pathogenic through the combination of ClinVar database, *in silico* prediction and gnomAD.

**Table 3:** The *FANCC* variants among familial breast and/or ovarian cancer patients (*n* = 255), sporadic breast cancer patients (*n* = 250) and female non-cancer controls (*n* = 248).

**cDNA change**	**AA change**	**Location**	**dbSNP ID**	**ClinVar**	**FBOC (%)**	**SBC (%)**	**FNCC (%)**	P^*^-value	**SIFT**	**PolyPhen-2**	**Provean**	**Panther**
c.51G>C	p.Q17H	Exon1	N/A	N/A	1 (0.4)	0	0	ND	TOLERATED	BENIGN	NEUTRAL	NEUTRAL
c.339G>A	p.W113X	Exon3	N/A	P/LP	1 (0.4)	0	0	ND				
c.758C>A	p.A253E	Exon7	N/A	N/A	1 (0.4)	0	0	ND	TOLERATED	POSSIBLY	DELETERIOUS	PROBABLY
										DAMAGING		DAMAGING
c.816C>T	p.I272I	Exon7	rs55719336	CIP	6 (2.4)	ND	ND					
c.903A>G	p.A301A	Exon9	N/A	N/A	1 (0.4)	ND	ND					
c.973G>A	p.A325T	Exon9	rs201407189	B/LB	8 (3.1)	9 (3.6)	5 (2.0)	0.231	TOLERATED	POSSIBLY	NEUTRAL	NEUTRAL
										DAMAGING		
c.1345G>A	p.V449M	Exon13	rs1800367	B/LB	1 (0.4)	ND	ND		DAMAGE	PROBABLY	NEUTRAL	PROBABLY
										DAMAGING		DAMAGING
c.1407G>A	p.T469T	Exon13	rs79722116	B/LB	6 (2.4)	ND	ND					

Numbering based on RefSeq: NM_000136; AA, amino acid; FBOC, familial breast and/or ovarian cancer; SBC, sporadic breast cancer; FNCC, female non-cancer controls;

* *Difference of frequencies between SBC and FNCC was compared by χ^2^ test; N/A, ID is not available in dbSNP for this variant; ND, not done; P, pathogenic; LP, likely pathogenic; CIP, conflicting interpretations of pathogenicity; B, benign; LB, likely benign*.

Two novel non-synonymous variants (c.51G>C, p.Q17H and c.758C>A, p.A253E) and one novel synonymous variant (c.903A>G, p.A301A) were identified in three unrelated patients, which were not reported in any public databases (Table 3). The patient with *FANCC* c.51G>C mutation was diagnosed with triple-negative (ER-, PR- and HER2-) breast cancer at the age of 41 years (Figure 2). She suffered from bone metastasis 3 years later after a modified radical mastectomy and died 6 years later. Familial clustering of tumors was also found, including ovarian cancer in her maternal grandmother, breast cancer in one of her maternal aunts, and liver cancer in another maternal aunt. In our case-control cohort, c.51G>C (p.Q17H) was not found in neither 250 SBC patients nor 248 FNCCs. *In silico* analysis suggested that this variant tended to be benign. Moreover, IHC analysis showed that FANCC was positive in this patient (Figure 2). The other non-synonymous variant c.758C>A (p.A253E) was found in another breast cancer patient who was diagnosed at 42 years old, with ER and PR positive, and HER2 negative. Her sister and paternal sister were diagnosed with breast cancer at 54 and 52 years old, respectively. Unfortunately, breast cancer tissues from this patient and blood samples from her sisters were not available for the further analysis. In our case-control cohort, c.758C>A was found in neither 250 SBC patients nor 248 FNCCs. As for *in silico* analysis, three algorithms suggested that this variant tended to be deleterious while the SIFT algorithm predicted it to be benign.

Among 255 FBOC patients, four SNPs (rs55719336, rs201407189, rs1800367, and rs79722116) were found with variant frequencies (Table 3). Two SNPs (rs201407189 and rs1800367) were non-synonymous (c.973G>A, p.A325T, and c.1345G>A, p.V449M) and the other two (rs55719336 and rs79722116) were synonymous (c.816C>T, p.I272I and c.1407G>A, p.T469T). The frequency of SNP rs201407189 (corresponding to *FANCC* c.973G>A) was similar in the aforementioned 250 SBC patients and 248 FNCCs (3.6 vs. 2.0%, respectively, *P* = 0.231). Although some algorithms predicted that p.A325T (rs201407189) and p.V449M (rs1800367) were likely associated with the protein function damage, these two SNPs (rs201407189, rs1800367) and rs79722116 were classified as benign or likely benign in ClinVar database. However, the synonymous variant (c.816C>T, rs55719336) was classified as conflicting interpretations of pathogenicity in ClinVar database. Actually, most of the submitters classified it as benign or likely benign, except one submitter who classified it as uncertain significance.

## Discussion

In this study, we identified one rare *FANCC* deleterious mutation (c.339G>A, W113X) in one patient (0.4%) by screening the germline mutations of *FANCC* gene in 255 *BRCA1/2*-negative Chinese women with FBOC. To our knowledge, this is the first report of a *FANCC* deleterious mutation in Chinese population which might have a distinct genetic landscape of breast cancer compared to the Caucasian population. We also identified two novel non-synonymous variants (c.51G>C, p.Q17H and c.758C>A, p.A253E), one novel synonymous variant (c.903A>G, p.A301A), two non-synonymous SNPs rs201407189 (c.973G>A, p.A325T) and rs1800367 (c.1345G>A, p.V449M), and two synonymous SNPs rs55719336 (c.816C>T, p.I272I) and rs79722116 (c.1407G>A, p.T469T).

FA is an autosomal recessive syndrome characterized as progressive bone marrow failure, congenital abnormalities and susceptibility to cancer. To date, 16 genes have been identified to be associated with FA (13). FA proteins work together with BRCA2/Rad51-mediated homologous recombination in double-stranded DNA repair (14). Biallelic inactivations of *BRCA2* and *BRCA1* can cause FA complementation group D1 (15) and a new FA subtype (16), respectively. Increasing evidence showed that breast cancer and FA share some susceptibility genes depending upon the monoallelic mutation or biallelic mutation. Recently, the FA susceptibility genes *FANCJ/BRIP1* (17), *FANCN/PALB2* (18), *FANCO/RAD51C* (19), *FANCP/SLX4* (20), and *FANCM* (21) have also been identified as breast cancer susceptibility genes.

Homozygous mutations in *FANCC* are responsible for FA complementation group C. FANCC is an essential substrate for forming a ternary complex together with FANCE and FANCD2, which is responsible for the FA DNA damage response pathway (22). Poor survival in breast cancer patients with alternative *FANCC* genes suggested that *FANCC* is a breast cancer suppressor (23). A study investigated the risk of cancer among FA gene heterozygous carriers, which recruited 944 relatives (784 grandparents and 160 other relatives) of FA probands from 312 families. The results showed that breast cancer, but not other cancers, had a significantly higher rate among carriers' grandmothers (SIR: 1.7; 95% CI: 1.1–2.7). Moreover, grandmothers who were *FANCC* mutation carriers had the highest risk of breast cancer (SIR: 2.4; 95% CI: 1.1–5.2) (24). However, another study which recruited 42 Ashkenazi Jewish women who were *FANCC* heterozygous carriers showed that the risk of breast cancer was not significantly elevated in the carriers (both 2.2% for the carriers and controls) (25). Additionally, the number of cancer cases among families with FA carriers were significantly fewer than those in the controls (25). These inconsistent results between studies might be related to the small sample size or multiple comparisons and the younger age of *FANCC* carriers.

The frequency of *FANCC* mutations in high-risk breast cancer patients has been under-investigated. The first study investigating *FANCC* germline mutations enrolled 88 *BRCA1/2*-negative familial breast cancer patients from the United Kingdom, finding no deleterious mutations by conformation sensitive gel electrophoresis followed by a sequencing assay (26). In 2012, Thompson et al. (5) identified the first *FANCC* deleterious mutations in breast cancer families, with the frequency of *FANCC* mutations of 0.7% (3/453) in their cohort of *BRCA1/2*-negative familial breast cancer patients, indicating that *FANCC* germline mutations in high-risk breast cancer patients would be very rare. Recently, three studies which were conducted in the United States (27), Russia (28), and China (29) used gene panel sequencing to screen the multi-gene germline mutations in high-risk *BRCA1/2*-negative breast cancer patients, yet none of the *FANCC* germline mutations were found. In our cohort of 255 *BRCA1/2*-negative FBOC patients, the frequency of *FANCC* deleterious mutations was 0.4%, which was comparable to the result reported in the Caucasian population. Since FA is very rare in the Chinese population, the mutation frequency and spectrum of FA genes remain unknown.

*FANCC* c.339G>A (W113X) was classified as pathogenic/likely pathogenic in ClinVar database while it was not reported in breast cancer patient. This patient had neither small mutations nor LGRs in *BRCA1/2* genes. The germline mutations of 98 genes including 24 known and candidate breast cancer susceptibility genes were screened by a gene panel sequencing assay, and no pathogenic or likely pathogenic mutation was found except *FANCC* c.339G>A. Moreover, IHC analysis showed that FANCC was negative in the breast cancer tissue of this patient. These results suggested that *FANCC* c.339G>A was a breast cancer susceptibility mutation.

## Conclusion

In conclusion, we have identified, for the first time, a *FANCC* deleterious mutation in one *BRCA1/2*-negative Chinese FBOC patient. Although this *FANCC* germline mutation was rare, it might have important clinic implications since *FANCC* is a breast cancer suppressor and is responsible for FA complementation group C. The number of identified genes responsible for moderate to high-risk susceptibility of breast cancer is increasing, which can account for ~ 1% of the affected families. Further investigations on the penetrance and spectrum of *FANCC* mutations are highly warranted for the genetic counseling among Chinese FBOC patients.

## Clinical Practice Points

1. The genetic etiology in more than 80% of Chinese women with high-risk breast cancer remains unknown. Increasing evidence has shown that breast cancer and Fanconi anemia (FA) share some susceptibility genes depending upon the monoallelic mutation or biallelic mutation.
2. Homozygous mutations in FANCC are responsible for FA complementation group C. FANCC is a breast cancer suppressor (22). Heterozygous FA gene carriers had a significantly higher risk of breast cancer among carriers' grandmothers (SIR: 1.7; 95% CI: 1.1–2.7). Moreover, grandmothers who were FANCC mutation carriers had the highest risk of breast cancer (SIR: 2.4; 95% CI: 1.1–5.2) (23). The study conducted by Thompson et al. (5) showed that FANCC was a novel breast cancer susceptibility gene in the Caucasian population, with the mutation frequency of 0.7% (3/453). Our study provided evidence, for the first time, that the FANCC deleterious mutation exists in Chinese familial breast cancer patients, with a frequency of 0.4% in our cohort.
3. Recently, genetic testing using gene panel sequencing assay and subsequent counseling for cancer (such as breast cancer) in high-risk populations has become more and more popular in China. However, researchers and clinicians might use different gene panels. Since FANCC deleterious mutations existed in Chinese high-risk breast cancer patients according to our data, genetic counseling for breast cancer shall also include FANCC deleterious mutations, though further investigations on the penetrance and spectrum of FANCC mutations are highly warranted.

## Author Contributions

W-MC is responsible for the study concept and design. W-MC, X-JW, and TC obtained funding. Z-WP and W-MC acquired data. Z-WP, X-WD, XJ, YG and W-MC analyzed and interpreted data. W-JM, YH, and C-JL collected the patients' samples and clinic data. W-MC and X-JW drafted the manuscript, and all authors revised it for important intellectual content. W-MC is the guarantor of this work.

### Conflict of Interest Statement

The authors declare that the research was conducted in the absence of any commercial or financial relationships that could be construed as a potential conflict of interest.

## Supplementary Material

Supplementary Table 1 Germline variants of 98 genes in the patient with *FANCC* deleterious mutation (c.339G>A, p.W113X).

## Abbreviations

FA: Fanconi anemia
SNPs: single nucleotide polymorphisms
LGRs: large genomic rearrangements
PCR: polymerase chain reaction
MLPA: multiplex ligation-dependent probe amplification
HGVS: Human Genome Variation Society
LOVD: Leiden Open Variation Database 3.0
ACMG: American College of Medical Genetics
OMIM: Online Mendelian Inheritance in Man
AA: Amino Acid
FBOC: familial breast and/or ovarian cancer
SBC: sporadic breast cancer
FNCC: female non-cancer control
IHC: immunohistochemistry
ER: estrogen receptor
PR: progesterone receptor
HER2: human epidermal growth factor receptor 2
FISH: fluorescence *in situ* hybridization.
